# Design and Exploratory Neuropharmacological Evaluation of Novel Thyrotropin-Releasing Hormone Analogs and Their Brain-Targeting Bioprecursor Prodrugs 

**DOI:** 10.3390/pharmaceutics5020318

**Published:** 2013-05-22

**Authors:** Katalin Prokai-Tatrai, Vien Nguyen, Szabolcs Szarka, Krisztina Konya, Laszlo Prokai

**Affiliations:** 1 Department of Molecular Biology and Immunology, University of North Texas Health Science Center, 3500 Camp Bowie Boulevard, Fort Worth, TX 76107-2699, USA; E-Mails: vien.nguyen@unthsc.edu (V.N.); szabolcs.szarka@unthsc.edu (S.S.); laszlo.prokai@unthsc.edu (L.P.); 2 Department of Pharmaceutical Sciences, UNT System College of Pharmacy, University of North Texas Health Science Center, 3500 Camp Bowie Boulevard, Fort Worth, TX 76107-2699, USA

**Keywords:** bioprecursor prodrug, brain-targeting delivery, thyrotropin-releasing hormone, synthetic peptide analog, analeptic effect, antidepressant-like activity

## Abstract

Efforts to take advantage of the beneficial activities of thyrotropin-releasing hormone (TRH) in the brain are hampered by its poor metabolic stability and lack of adequate central nervous system bioavailability. We report here novel and metabolically stable analogs that we derived from TRH by replacing its amino-terminal pyroglutamyl (pGlu) residue with pyridinium-containing moieties. Exploratory studies have shown that the resultant compounds were successfully delivered into the mouse brain after systemic administration via their bioprecursor prodrugs, where they manifested neuropharmacological responses characteristic of the endogenous parent peptide. On the other hand, the loss of potency compared to TRH in a model testing antidepressant-like effect with a simultaneous preservation of analeptic activity has been observed, when pGlu was replaced with trigonelloyl residue. This finding may indicate an opportunity for designing TRH analogs with potential selectivity towards cholinergic effects.

## 1. Introduction

**Figure 1:**
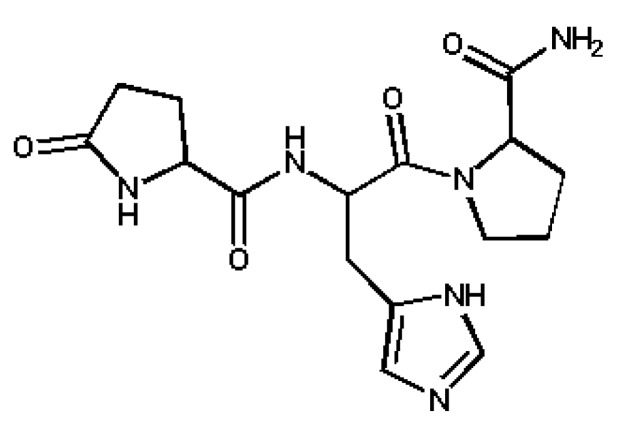
Chemical structure of thyrotropin-releasing hormone (TRH, pGlu-His-Pro-NH_2_).

Our laboratory has been involved in medicinal chemistry-driven research with attention to facilitating drug delivery of central nervous system (CNS) agents via prodrug approaches. Thyrotropin-releasing hormone (TRH), a small peptide (pGlu-His-Pro-NH_2_, Figure 1), has been one of the main focuses in this regard [1,2,3,4,5]. TRH was the first hypothalamic factor characterized [6] and it has also served as a lead structure for CNS drug discovery [7,8], due to its multitude of central actions [9,10,11]. These actions are independent of the hypothalamic-pituitary-thyroid axis; thus, the peptide can also function as a neuromodulator through various neurotransmitters, most prominently via acetylcholine [10].

The use of TRH as a neuropharmaceutical agent has been hampered by, e.g., its inadequate metabolic stability [12], poor CNS bioavailability [13] and, therefore, profound endocrine activity due to high systemic doses needed for establishing therapeutic concentration in the brain. Analogs preserving the central benefits of TRH and possessing enhanced *in vivo* metabolic stability may, however, overcome these limitations [3,11,14,15,16]. Nevertheless, the blood-brain barrier (BBB) has been an obstacle to drug delivery, even with metabolically stable analogs of the peptide [17]. As it has been well-documented, the BBB prevents the passive transport of the overwhelming majority of chemical entities into the brain from circulation [18]. Only a limited number of small molecules with suitable physicochemical properties can reach this organ from the blood at adequate concentration in the absence of specific transporters. Hence, CNS drug delivery has been a challenging endeavor, especially for peptides [19].

Various invasive and non-invasive approaches have been conceived to usher drugs into the brain from the circulation by essentially tricking the BBB [20,21,22]. Among the non-invasive methods, the prodrug approach offers a viable option for CNS-drug delivery of small- and medium-sized neuropeptides [5]. By definition, a prodrug is an inert precursor of the active agent (parent drug) that remains inactive until specific enzyme(s) liberates the parent drug *in vivo*. Prodrugs are synthesized from the parent agents by transient chemical modification(s), such as esterification [23]. Because lipophilicity is one of the major governing factors for passive transport across the BBB [24], enhancing this physicochemical property via prodrug creation has been particularly useful for peptides that are generally highly hydrophilic substances. In most cases, the functional group(s) in a peptide sequence (e.g., -NH_2_, -COOH or -OH) is derivatized not only to enhance lipophilicity, but also to render the peptide “neutral” at physiological pH to favor diffusion through the BBB [25]. The chemically introduced “promoiety(ies)”, whose presence results in the loss of the innate activity of the parent agent, is metabolically and/or chemically labile. Therefore, removal of the promoieties unmasks the active agent.

In another prodrug approach that leads to the so-called bioprecursor prodrugs [26], no auxiliary “promoiety” is attached to the parent drug, because a bioreversible chemical manipulation (e.g., reduction or oxidation) is carried out within the drug molecule itself [2,3]. We have applied this particular prodrug methodology with encouraging results to generate centrally active and non-endocrine TRH analogs upon replacement of the central basic histidyl residue (His) in the TRH sequence with amino acids having a pyridinium-containing side chain [2,3]. These agents are metabolically stable, as replacement of His eliminates the TRH-degrading ectoenzyme-sensitive pGlu-His bond mostly responsible for the very short biological half-life of TRH in the blood [8,12,27]. An additional rationale for replacing the central residue has been to abolish (or at least diminish) the endocrine effects seen with TRH [15,27]. Concurrently, the pyridinium moiety of the new central residue can easily be converted *via* chemical reduction to a dihydropyridine [2,3,4,28], and the resultant neutral peptides can serve as bioprecursor prodrugs of these TRH analogs [2,3]. The preferential activation of the prodrug to the permanently charged parent agent in the brain occurs via oxidation, analogously to that of the endogenous NADH → NAD^+^ reaction [28]. At the same time, the “oxidized prodrugs” (*i.e.*, the actual pyridinium-type TRH analogs) should quickly be eliminated from the periphery, due to their ionic nature [28], while oxidation of the prodrug in the brain actually prevents efflux from the BBB. Therefore, this particular prodrug approach is expected to result in brain-enhanced drug delivery.

*In vivo* validation of this design was done by utilizing typical and convenient TRH-associated central activities; specifically, the analeptic [29,30] and antidepressant-like effects [31,32]. The latter is monitored by a swim test introduced by Porsolt *et al.* [33]. The antagonism of barbiturate-induced anesthesia (*i.e.*, an analeptic response) has been the most frequently used paradigm to indicate the extent of the activation of cholinergic neurons [34] and, thus, a successful central delivery of TRH and related agents. One of the analogs we created by His replacement with a pyridinium-containing residue produced TRH-equivalent analeptic and antidepressant-like responses upon intravenous (*i.v.*) administration of its brain-targeting dihydropyridine-type of bioprecursor prodrug [2,3], thereby indicating not only a successful brain delivery of the target TRH analog, but also its ability to retain the neuropharmacological responses typical of TRH.

Next, we probed whether [Glu^2^]TRH, pGlu-Glu-Pro-NH_2_, a non-endocrine and metabolically stable TRH-related peptide that possesses numerous central TRH actions [30,34,35,36], could also be utilized for the same design concept to obtain its pyridinium-containing analogs suitable for convenient bioprecursor prodrug preparation. In [Glu^2^]TRH, we replaced the *N*-terminal pGlu with trigonelloyl residue [37] based on findings that the therapeutically most successful TRH analogs have been derived by pGlu-replacement [38]. Indeed, when the dihydropyridine-based bioprecursor of this agent that was also “lipidized” as hexyl ester on the central Glu to supply adequate lipophilicity and neutral character to the prodrug [36], administered via *i.v.* to mice, we recorded a statistically significant antidepressant-like response; yet, this peptide significantly underperformed both TRH and [Glu^2^]TRH in the analeptic test. We then reasoned that replacement of pGlu with a pyridinium moiety in TRH may also allow for improving selectivity towards the antidepressant-like property and, thus, potentially leading to templates useful for designing a novel class of antidepressants.

**Figure 2:**
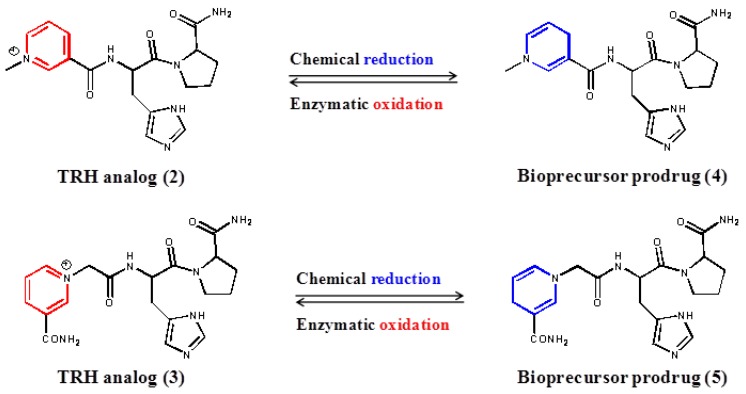
Chemical structures of novel TRH analogs (**2** and **3**) and their brain-targeting bioprecursor prodrugs (**4** and **5**, respectively), as well as schematic illustration of prodrug synthesis from and their bioactivation to the respective TRH analogs.

Altogether, based on our previous findings [2,3,4,5,37], in the present study, we explored whether replacement of pGlu with pyridinium-based residues in the TRH sequence itself (Figure 2) would also lead to analogs with improved selectivity towards the antidepressant-like effect over the analeptic response. In exploratory proof-of-concept studies, we monitored these neuropharmacological measures upon systemic administration of the bioprecursor prodrugs of the novel TRH analogs having a permanently charged *N*-terminus.

## 2. Experimental Section

### 2.1. Materials

Reagents and solvents used in the study were purchased from Sigma-Aldrich (St. Louis, MO, USA) and were of reagent grades. Pre-loaded (0.48 mmol/g) Fmoc-Pro-Rink Amide-MBHA resin was from Anaspec Inc. (Fremont, CA, USA).

### 2.2. Animals

Male Swiss-Webster mice (30 ± 2 g body weight) used for monitoring the typical TRH-related analeptic and antidepressant-like effects were obtained from Charles River Laboratories (Wilmington, MA, USA). All procedures were reviewed and approved by the institutional animal care and use committee before initiation of the studies. Animals were housed four per cage with *ad libitum* access to water and food in a room where temperature is kept between 21 and 23 °C with a normal day/night cycle. Each animal was tested only once.

### 2.3. Synthesis of Test Compounds

Solid-phase peptide synthesis (SPPS) utilizing standard 9-fluorenylmethyloxycarbonyl (Fmoc)-based chemistry was employed to assemble TRH analogs **2** and **3** (Figure 2), as reported before [2,3,4,5,16,37]. Briefly, we used a preloaded Fmoc-Pro-Rink Amide-MBHA resin, 20% (*v*/*v*) piperidine in *N*,*N*-dimethylformamide (DMF) for deprotection and DMF and methanol for washing. Coupling was enforced with (benzotriazol-1-yloxy)tripyrrolidinophosphonium hexafluorophosphate (PyBOP) in the presence of a *N*,*N*-diisopropylethylamine (DIPEA) and hydroxybenzotriazole (HOBt) using amino acid:PyBOP:DIPEA:HOBt, 1:1:2:1 molar ratio. The peptide chain was terminated with trigonellic acid (1-methylpyridinium-3-carboxylate hydrochloride) for analog **2** or was further extended with Fmoc-Gly. In the latter case, a solid phase Zincke reaction [37] was done to obtain **3**. The novel chemical entities (**2** and **3**) were cleaved from the resin with trifluoroacetic acid/water 98:2 (*v*/*v*) and purified by semi-preparative reversed-phase high-performance liquid chromatography (RP-HPLC). For the preparation of the bioprecursor prodrugs **4** and **5** (Figure 2), the TRH analogs (**2** and **3**) were reduced to the corresponding neutral dihydropyridines (**4** and **5**) with a well-established and straightforward method using sodium dithionite [2,3,28,37,39]. The identity and purity of the test agents were confirmed by liquid chromatography-mass spectrometry (LC-MS) and analytical RP-HPLC. Detailed analytical characterizations, including NMR spectral assignments, will be included in a subsequent publication.

### 2.4. *In Vitro* Metabolic Stability

Freshly made mouse brain homogenate and plasma were used for these studies, as reported before [3,4,5,16]. Briefly, immediately following removal, the brain was weighed and transferred to a Potter-Elvehjem tissue grinder (Wheaton, Millville, NJ, USA) on ice bath. Twenty-percent (*w*/*v*) homogenates were prepared in ice-cold, pH 7.4 phosphate-buffered saline. Plasma was prepared by centrifugation at 1500 rpm for 10 min using a Sorwal (Newtown, CT, USA) GLC-1 general laboratory centrifuge. The brain homogenate and plasma was used immediately for stability studies. Approximately 100 nmol of test compound was used in 1 mL plasma or brain homogenate, the mixture was incubated at 37 °C in a temperature-controlled, shaking water bath. Aliquots (100 μL) were removed after 2, 5, 15, 30, 60, 120 and 180 min of incubation and transferred into a 1.5 mL Eppendorf tube containing 200 μL of ice-cold solution of 5% (*v*/*v*) acetic acid in acetonitrile. The samples were centrifuged, and the supernatant was removed and analyzed by HPLC to monitor the decline in the concentration of the analyte added. HPLC analyses were performed on an Agilent 1100 HPLC system (Agilent Technologies, Santa Clara, CA, USA) equipped with a UV-Vis detector and operated at 254 nm. Separation was carried out on a Phenomenex (Torrance, CA, USA) Aeris PEPTIDE XB-C18 column (150 × 2.10 mm *i.d.*, 3.6 µm particle size) at ambient temperature with a 0.4 mL/min flow rate. The eluent consisted of (A) 0.5% trifluoroacetic acid in water (*v*/*v*) and (B) 0.05% trifluoroacetic acid in acetonitrile (*v*/*v*).

### 2.5. Analeptic Effect

These studies were conducted according to our established procedure [1,2,3,4,5,16,37]. Briefly, mice were divided into groups of *n* = 10. Test compounds were dissolved in saline. The vehicle alone (30 µL; control group) or equimolar doses of test compounds, including TRH (10 μmol/kg body weight), were injected through the tail vein of mice. After 10 min, each animal received an intraperitoneal (*i.p.*) injection of sodium pentobarbital solution at a dose of 60 mg/kg body weight. The sleeping time was recorded from the onset of loss of the righting reflex until the reflex was regained. The two trained observers recorded the sleeping times independently from each other and were unaware of the treatment regimen.

### 2.6. Porsolt’s Swim Test (PST) to Assess Antidepressant-Like Activity

Behavioral studies for antidepressant-like activity were conducted with a validated model, as reported before [16,37,39,40]. Test compounds were administered *i.v.* (30 µL volume) through the tail vein at the dose of 3 µmol/kg body weight. The control group received the saline vehicle (30 µL) only. Briefly, for 6 min, the immobility time (s), the duration of motionless floating after the cessation of struggling and making only movements necessary to keep the head above the water was recorded simultaneously by two trained observers that were blinded to the treatment protocol. Immobility is considered to be “depressed”; therefore, test compounds are deemed to elicit antidepressant-like activity upon producing significantly shorter immobility time than the vehicle control.

### 2.7. Data Analysis

Data are expressed as the mean ± SEM, and statistical evaluations were done by one-way ANOVA. Two-group comparisons employed Dunnett’s or Fisher’s PLSD *post hoc* tests when a significant omnibus ANOVA was found (α = 0.05 two-tailed), noting that type-I error correction is not necessary with orthogonal planned comparisons [41].

## 3. Results and Discussion

The synthesis of target compounds with pyridinium-based *N*-termini (**2** and **3**, as shown in Figure 2) via SPPS proceeded smoothly, as our laboratory has been routinely doing such types of preparative works [1,2,3,4,5,16,25,36,37]. *In vitro* stability studies in freshly made mouse brain homogenate and plasma [3,4,5,16], respectively, showed that **2** and **3** were metabolically stable (data not shown); no significant degradations of the compounds were detected within 2 h by RP-HPLC. This finding was expected [27], since the hydrolysis-sensitive pGlu-His bond [12] was eliminated when we replaced pGlu with pyridinium-containing (and also unnatural) residues. Previously, we have also confirmed that when the central His is replaced in the TRH sequence with pyridinium-based amino acids, the resultant TRH analogs were metabolically stable [2,3]. In comparison, TRH’s half-life is around 10 min in mouse plasma and 15 min in mouse brain homogenate [3]. Additionally, we did not find significant differences in the *in vitro* metabolic oxidation, *i.e.*, bioactivation of the bioprecursor prodrugs **4** and **5**, to the corresponding TRH analogs (**2** and **3**, respectively, Figure 2) in the selected biological media. These findings are comparable to those we have reported before for the dihydropyridine type of prodrugs [2,3,28,37]. Accordingly, the dihydropyridine→pyridine oxidation (*i.e.*, prodrug to TRH analog conversion) occurred with t_½_ of about 5 min in mouse brain homogenate, and around 22 min t_½_ values were determined in mouse plasma (data not shown). The increased resilience of **4** and **5** against oxidation in the plasma *versus* brain should be beneficial for BBB transport upon their systemic administrations. We also did not find significant differences in these measures between **4** and **5.** It should be noted that the *in vitro* oxidation of this type of unsubstituted dihydropyridines in the selected biological matrices apparently does not significantly depend on the rest of the chemical structure of the prodrugs [28].

**Figure 3:**
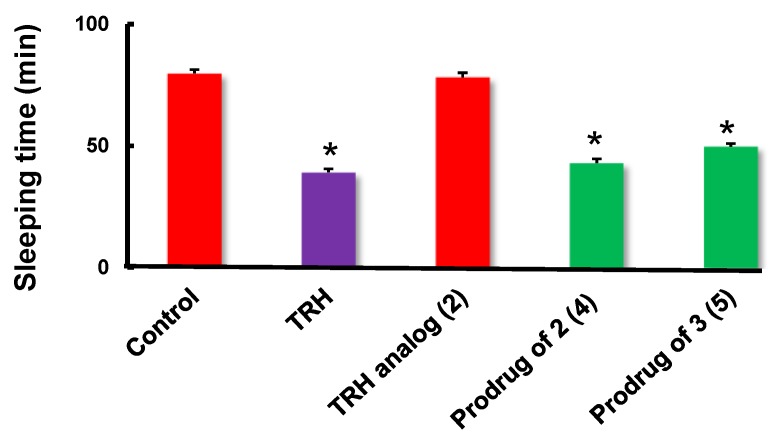
Reversal of pentobarbital-induced sleeping times (*i.e.*, analeptic effect) in mice after administration of TRH, TRH analogue **2** and bioprecursor prodrugs **4** and **5**. Sleeping time is expressed as the mean ± SEM (*n* = 10 per group). Ten min after *i.v.* injection of the test compound at a dose of 10 μmol/kg body weight, pentobarbital (60 mg/kg, *i.p.*) was injected. Sleeping time was recorded from the onset of the loss of the righting reflex until the reflex was regained. * Statistical significance determined using analysis of variance (ANOVA) followed by *post hoc* Fisher’s PLSD test for multiple comparisons: *p* < 0.05 *versus* vehicle control.

*In vivo* validation of brain-targeting of **2** and **3** via their bioprecursor prodrugs, **4** and **5**, respectively (Figure 2), was done by utilizing two well-established neuropharmacological paradigms based on typical TRH-related central actions. Specifically, the analeptic model [29,30] and PST [31,32] were used for these studies. The analeptic effect is surveyed in a simple paradigm by measuring the reversal of barbiturate-induced sleeping time in mice [5,7,30]. In PST, after mice give up swimming in a controlled environment consisting of a water tank, the floating time is recorded within a fixed time period, as floating is believed to correspond to a “depressive mood” [33]. We used TRH as a positive control at equimolar concentration (10 μmol/kg body weight) in these studies. The negative control group received the vehicle only. With exploratory proof-of-concept experiments summarized in Figure 3, we have shown so far that, for example, analog **2*** per se* did not elicit an analeptic response, due to its ionic nature (*i.e.*, having a pyridinium moiety) that prevented its passive transport across the BBB from the blood after *i.v.* injection.

On the other hand, administration of the corresponding bioprecursor prodrug (**4**) produced a statistically significant reversal of pentobarbital-induced sleeping time; moreover, this analeptic response was statistically not different from that of TRH under the experimental conditions applied. These data imply that (i) the prodrug (**4**) successfully passed the BBB from the circulation and, (ii) once in the brain it converted to **2** via oxidation (Figure 2) that, (iii) in turn, elicited the desired TRH-characteristic neuropharmacological response.

A slightly longer sleeping time was detected upon administration of **5**, which is the bioprecursor prodrug of **3**. In the latter, the pyridinium moiety is attached via a -CH_2_- linker that may result in more flexibility compared to **2**. Overall, the structural differences between **2** and **3** may indicate that replacement of pGlu with pyridinium-type moieties could be quite permissive in structural features to maintain analeptic response.

Similarly to the analeptic studies, analog **2** carrying a permanent positive charge failed to elicit an antidepressant-like effect in the exploratory PST studies upon systemic administration (Table 1). However, when it was injected to mice in its bioprecursor prodrug form (**4**, Figure 2), a statistically significant decrease in the immobility time (approximately 19%) was recorded compared to the saline-treated control group (arbitrarily taken as 100%). Nevertheless, analog **2** did still significantly underperform TRH (approximately a 36% decrease in the immobility time compared to control) in this paradigm, even though it produced a practically TRH-equivalent analeptic response upon brain-delivery via its prodrug (Figure 3, Table 1).

**Table 1:** Porsolt’s swim test (PST) immobility times reflecting the antidepressant-like effect of the TRH analog **2** with or without utilizing its bioprecursor prodrug **4**. TRH was used as a positive control. Test compounds were administered *i.v.* at the dose of 3 µmol/kg body weight. * *p* < 0.05 *versus* saline vehicle control (ANOVA followed by Dunnett’s test).

Test agent	Immobility time (s) mean ± SEM	% Change in immobility time compared to control ^a^	% Change in sleeping time compared to control ^a,b^
Saline	288 ± 6	0	0
TRH	184 ± 5 *	−36	−51 *
Analog **2**	279 ± 8	~0	~0
Prodrug of **2** (**4**)	231 ± 6 *	−19	−46 *

^a^ Control arbitrarily taken as 100%; percent decrease is calculated as [1 − (*X*_test agent_/*X*_control_)] × 100, where *X* denotes the given neuropharmacological response; ^b^ based on analeptic data shown in Figure 2.

These preliminary findings are in sharp contrast with our original expectation; *i.e.*, we anticipated that replacement of pGlu with trigonelloyl residue in TRH would also result in enhanced antidepressant-like activity over analeptic response, as seen with the corresponding [Glu^2^]TRH analog [37]. The latter peptide elicited a significantly improved selectivity towards the antidepressant-like effect, compared to that of TRH and [Glu^2^]TRH. Apparently, our data presented here indicate for the first time that the TRH-characteristic analeptic and antidepressant-like activities may be dissociated upon designing appropriate TRH analogs. This type of agent may also overcome drug delivery issues in preclinical studies by using the corresponding dihydropyridines, as brain-targeting bioprecursor prodrugs.

## 4. Conclusions

Our exploratory study has shown for the first time that replacement of the *N*-terminal pGlu of TRH with pyridinium-type moieties maintains the pharmacological activities of the resultant peptide analogs in mice. However, the loss of potency in the model testing antidepressant-like effects with a simultaneous preservation of analeptic potency has been apparent, which may indicate an opportunity for the discovery of TRH analogs with potential selectivity towards cholinergic effects. In addition, we have demonstrated, again, the value of a bioprecursor prodrug approach to facilitate brain delivery of molecules containing pyridinium-type moieties. Collectively, these encouraging data warrant further investigations, including addressing potential endocrine responses elicited by the TRH analogs reported in this communication.
